# Massive seafloor mounds depict potential for seafloor mineral deposits in the Great South Basin (GSB) offshore New Zealand

**DOI:** 10.1038/s41598-021-88620-x

**Published:** 2021-04-28

**Authors:** Omosanya Kamaldeen Olakunle, Lawal Muhedeen Ajibola, Iqbal H. Muhammad, Yizhaq Makovsky

**Affiliations:** 1 Oasisgeokonsult, 7052 Trondheim, Norway; 2 Department of Geoscience and Petroleum, Norwegian University of Science and Technology, Trondheim, Norway; 3 Dr Moses Strauss Department of Marine Geosciences, University of Haifa, Haifa, Israel; 4 Department of Earth and Environmental Sciences, Bahria University, Islamabad, Pakistan

**Keywords:** Geology, Geophysics, Mineralogy, Tectonics, Volcanology

## Abstract

Seafloor mounds are enigmatic features along many continental margins and are often interpreted as gas hydrate pingoes, seep deposits, mud volcanoes, or hydrothermal mounds. When such mounds occur in basins with past volcanic activities, they have the potential to host seafloor metalliferous deposits, which is generally overlooked. Using geophysical datasets, we document the fluid plumbing systems that promoted the formation of seafloor mounds in the Great South Basin (GSB), offshore New Zealand. We also investigate these mounds as potential seafloor metalliferous deposits. Our results reveal 9 seafloor mounds (~ 137 m high) above gigantic (~ 5.4 km high) fluid escape pipes that are associated with deeper crystalline rocks. The structural make-up of the mounds, their geospatial relationships with the pipes and intrusive rocks, and geophysical properties suggest a primary volcanic or hydrothermal origin for the culpable fluids and mounds respectively. Fluids derived from deeper coal beds and shallow foraminiferal oozes in the basin constitute secondary fluid sources focused along polygonal faults and lateral flow cells. A main sub-vertical and minor lateral fluid plumbing patterns are proposed. The relationship between the mounds, pipes, underlying intrusive rocks, and upward routing of mineral-rich fluids could have implications for the formation of ore-grade mineral deposits on the seafloor in the GSB.

## Enigma of interpreting seafloor anomalies from seismic reflection data

Numerous modern seafloors are characterized by sites of focused fluid discharge. These sites are often associated with the release of deep or shallow hydrocarbon fluids, hydrothermal fluids, or pore water^1–4^. Where such seafloor sites are mounded, there is often no straightforward interpretation for the origin and nature of the mounds. However, direct seafloor observations and high-resolution geophysical imaging have assisted in identifying many seafloor mounds as gas hydrate pingoes with associated seep deposits or authigenic carbonate slabs that are often colonized by dense faunal communities^2,5,6^. Such mounds have also been interpreted as mud volcanoes in many cases^7,8^. With the availability of drilled samples, e.g. in the Faroe-Shetland Basin, offshore Scotland^9^ and the Vøring Basin, offshore Norway^10^, many buried mounds have also been interpreted as hydrothermal vent complexes that are associated with volcanism.

On seismic datasets, many seafloor mounds  have been observed to be linked to underlying high amplitude features (e.g. magmatic sills or dykes, etc.) and vertical zones of disturbed seismic reflections (conduits/fluid escape pipes), allowing a volcanic or hydrothermal origin to be inferred for the mounds^9,11^. Although, such seafloor mounds provide an avenue to study the interaction between the oceans and the subsurface sedimentary units, the geology associated with their formation and their potential for forming economic ore-grade metalliferous deposits is not well understood^12^. This work investigates the origin of nine seafloor mounds and the fluid plumbing systems associated with their formation. It also assesses their potential for forming deep-sea metalliferous deposits, using 2D seismic, gravity, magnetic and well log datasets from the Great South Basin (GSB), offshore New Zealand. The GSB, with up to 8 km of sedimentary fill^13,14^, associated past rifting, magmatic activity, structural deformation^15^, and widespread occurrence of fluid escape pipes and mounds^16^ presents an ideal location for this study. Today, deep-sea metalliferous deposits are receiving increasing global attention, owing to their potentially huge commercial values and geo-political and scientific importance^17^. For these reasons, there is growing importance in understanding the geological processes and fluid plumbing systems controlling the formation of mounds and their potential for forming metal-rich deposits in marine sedimentary basins.

### Geologic settings of the Great South Basin

**Figure 1:**
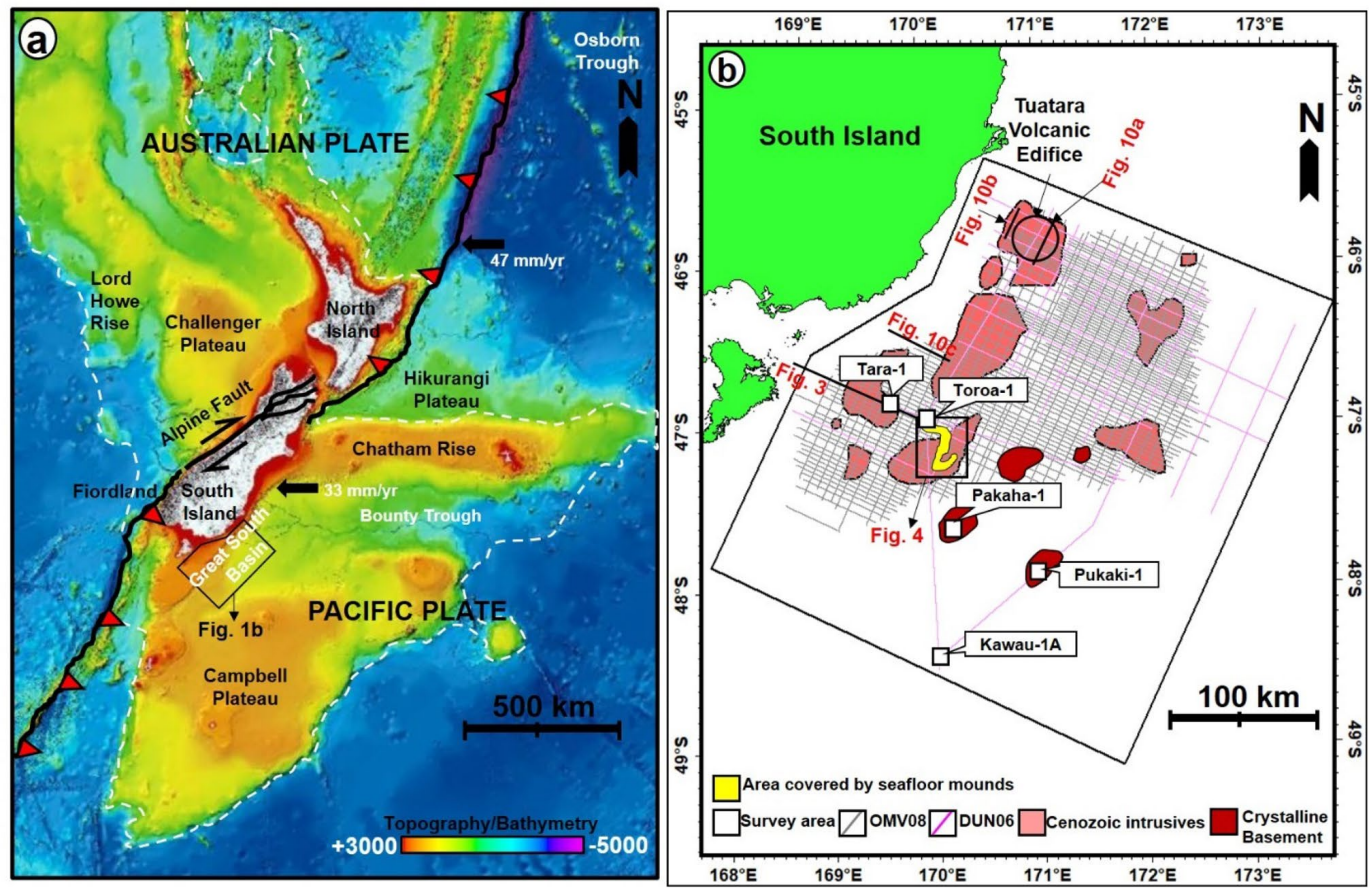
(**a**) 250 m gridded bathymetric and topographic map showing the structural elements of New Zealand and rough boundary of seismic surveys and study area in white rectangle. *The white dashed line defines the boundary of continent Zealandia*^85^*. The bathymetry map was downloaded from *https://niwa.co.nz/our-science/oceans/bathymetry (**b**) Map showing the outline of the two seismic surveys used for this study, including the locations of wellbores used for constraining the ages of the interpreted horizons. *Note: The small black rectangle shows the location of* Fig. 4a. The map in (**b**) is projected in WGS84 datum Geodetic.

The GSB is located at the southern margin of the South Island of New Zealand and in water depths of 300 m to 600 m of the modern shelf area (Fig. 1a). Structurally, the GSB lies to the south of the Stokes Magnetic Anomaly System (SMAS) and can be traced until a significant ENE-trending tectonic boundary called the Campbell Plateau (Fig. 1a). This structural boundary is defined by linear gravity and magnetic anomalies otherwise known as the Campbell Magnetic Anomaly System (CMAS)^18^. The geodynamic evolution of the Great South Basin is divided into syn-rift, post-rift, and syn-orogenic phases, which were synchronized with changes in the regional stress field and relative motion between the Australian, Pacific, and Antarctic plates^13,19^. The syn-rift phase (112–82 Ma) was dominated by horizontal extension while post-rift activities (82–19 Ma) include differential subsidence, compaction, tilting, and gravitational instabilities. During the Cretaceous, rifting involved separation of the Australian, Pacific, and Antarctica plates along a complex system of ridges and dispersal of the New Zealand micro-continent within the Pacific plate^20,21^. The basin framework inherited from the Cretaceous rifting, and basement faults, subsequently influenced younger tectonic activities such as compressional reactivation during the Eocene and Neogene shortening^15^. Post-rift relaxation of the GSB was accompanied by strong subsidence in the central part of the basin, localized uplift at previous rift shoulders, and tilting^22^. At the basin’s margin, vertical instability probably triggered slope instabilities and was accompanied by horizontal shortening and vertical uplift that were concomitant with the Kermadec subduction^20,22^ during the syn-orogenic phase (19 Ma-Recent). During this last phase of basin evolution, most of the inherited syn-rift normal faults were inverted^15^.

**Figure 2:**
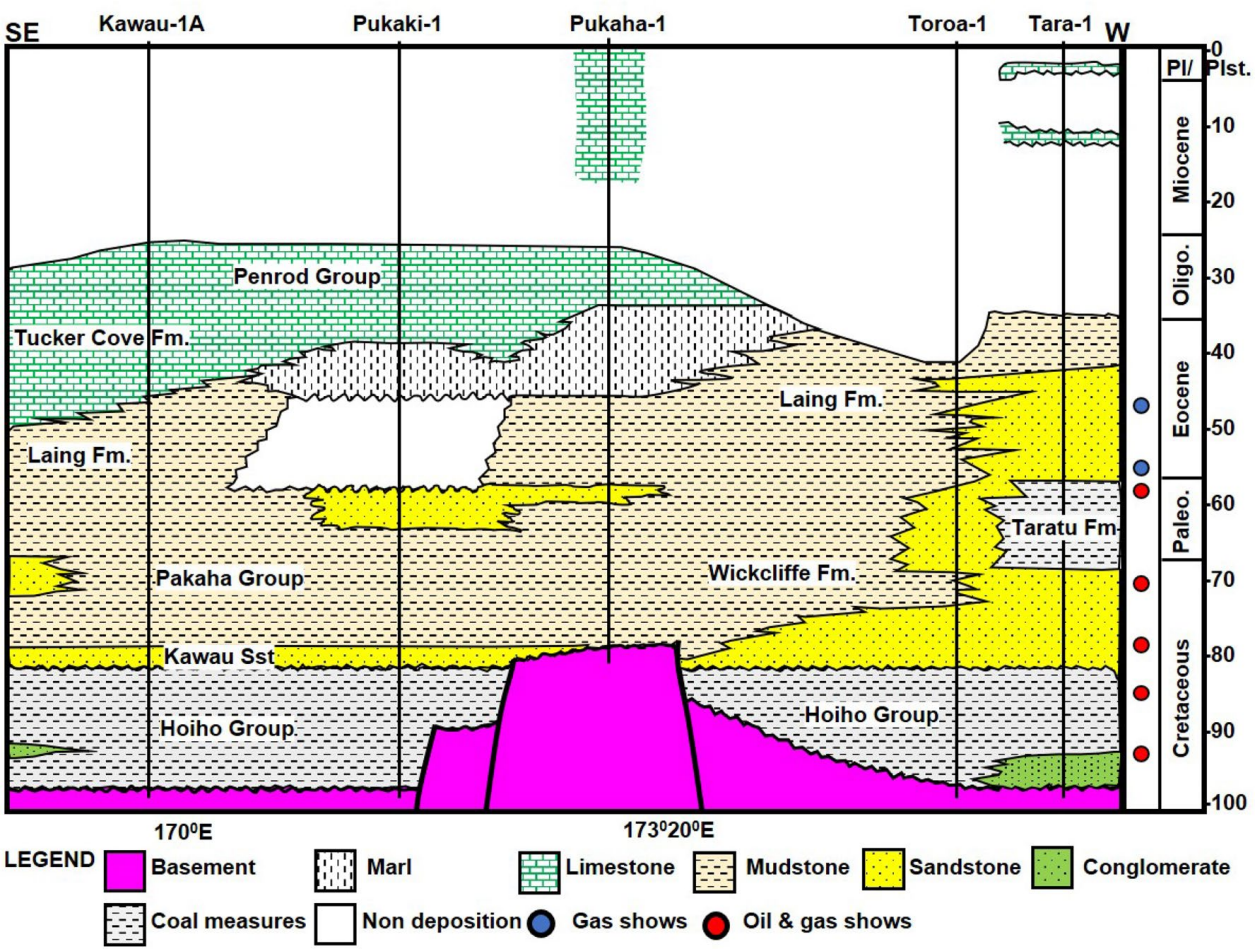
Lithostratigraphic correlation across all the wellbores available in the study area (Modified from New Zealand Petroleum and Minerals, 2014. Pl: Pliocene. Plst: Pleistocene).

The oldest rocks in the GSB are silicic to intermediate plutonic and metasedimentary rocks^23,24^, which are remnants of the Gondwanaland in the Early Geosynclinal Phase (Fig. 2). The Hoiho Group rocks are the oldest known sedimentary sequence in the basin, they are mid-Cretaceous in age and directly overlie the basement^25^. This group is composed of syn-rift deposits, such as non-marine conglomerates, sandstone, mudstone, and in some places, coal measures from fluvial and lacustrine environments^26^. By the Late Cretaceous, widespread fluvial systems that were generally flowing to the northeast had developed along the axes of the major grabens^27^. And with prolonged transgression and continued subsidence in the Late Cretaceous to Paleogene period, the region was drowned in an earlier terrestrial and near-shore environment^14^. The overlying Pakaha Group of Late Cretaceous to Palaeocene is characterized by widespread, fully marine facies followed by shallowing to coastal environments^27^.

The Pakaha Group consists of the Kawau, Wickliffe, Taratu, and Tartan Formations. In the eastern part of the GSB, the Hoiho Group is overlain by transgressive sandstones of the Kawau Formation, consisting of white to grey, coarse to fine-grained sandstones that were deposited from 84 to 80 Ma in a shallow marine environment^28^. The Kawau Formation marks the base of the Wickliffe Formation and Pakaha Group in wellbore Kawau-1A and Pukaki-1 (Fig. 2). Along the basin margin to the north and the northwest, non-marine, proximal sandstones of the Taratu Formation were deposited during the Campanian–Palaeocene interval^29^. The Taratu Formation overlies the Hoiho Group in the Tara-1 well (Fig. 2) and comprises of other rocks such as quartzose grit, conglomerates, and shales with coal measures. Unlike the Hoiho Group, the coal measure of the Taratu Formation had brackish water influx^30^. The Taratu is the equivalent of the Kawau sandstone in the northwestern part of the basin and changes from terrestrial to marine deposition, marking the contact with the Wickliffe Formation^13^.

Overlying the Kawau sandstone in the deeper parts of the basin is the Late Cretaceous to Palaeocene Wickliffe Formation, which primarily consists of marine shales and siltstones^31^. Near the transgressive coastline, the Wickcliffe Formation transits to shallow marine sandstone and organic-rich coastal plain sediments. Both at the base and the top, the Wickliffe Formation is bounded by unconformities that formed from 61 to 57 Ma^31^. However, the upper portions of the Wickliffe Formation contain the Late Palaeocene organic-rich mudstones of the Tartan Formation^13^. The Rakiura Group consists of fine-grained marine siliciclastic sediments and hemipelagic mudstones of the Laing and Tucker Cove Formations, which were deposited from 55.8 to 33.9 Ma^29^. Sedimentary successions deposited after the Rakiura Group from the Oligocene to the Recent, i.e. from 33.9 to 0 Ma, are deep-water pelagic marls of the overlying Penrod Group^29^. The Penrod Group contains thick mudstone sequences in the GSB, and hemipelagic mudstones with polygonal faults.

In terms of volcanism, widespread and long-lived intraplate magmatic and volcanic activities occurred across Zealandia following the breakup of Gondwana in the Late Cretaceous and persisted throughout the Cenozoic^32,33^. The major intraplate volcanism in the GSB are dated Late Cretaceous, Palaeocene to Early Eocene, Late Eocene to Early Oligocene and Middle to Late Miocene ^34^ and are depicted by several buried volcanic features including the Late Cretaceous to Early Eocene (~ 85–45 Ma) Tuatara Volcanoes^32^, the Upper Eocene to Lower Oligocene (40–34 Ma) Waiareka–Deborah volcanoes^35^, the Early Miocene (16–11.7 Ma) Dunedin Volcano ^35^ and references therein, the Miocene (12–6 Ma) Akaroa and Lyttleton volcanoes^34,35^, the Oligocene to Early Miocene Papatowai volcanoes^36^ and the Pleistocene Toroa volcanoes that have been observed in the shallow sediment column of the GSB^36^. Moreover, volcanic sequences representing Miocene volcanism have also been reported onshore^37^ and from an offshore well^38^ in the GSB, while Holocene volcanic activities were reported on the northern island of Zealandia^39^.

## Results

### Seismic stratigraphy of the study area

**Figure 3:**
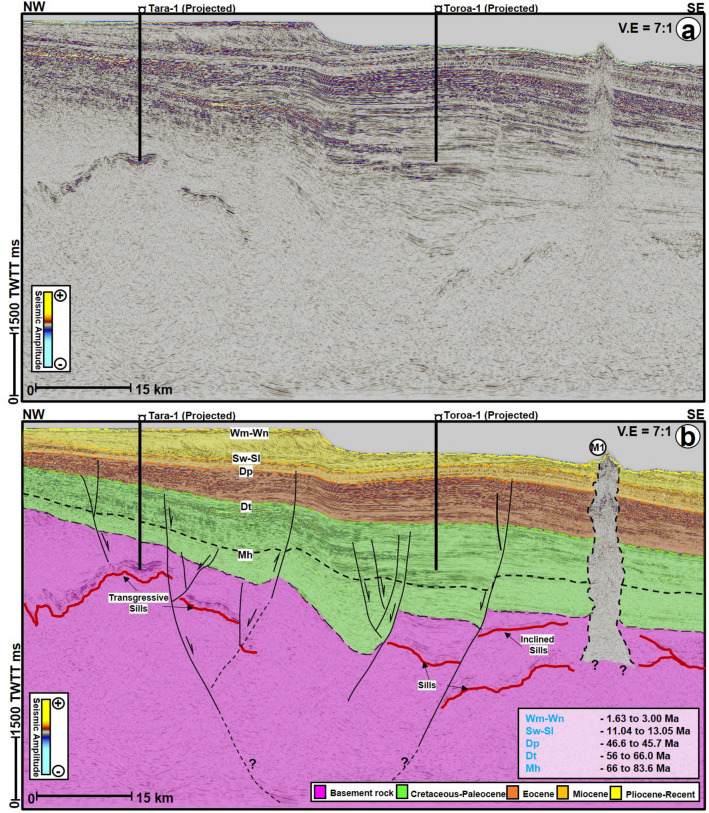
Uninterpreted (**a**) and interpreted (**b**) NW–SE seismic profiles showing the link between mound M1, its underlying fluid escape pipe, intrusive rocks, and the basement rocks of the study area. Intrusive rocks or magmatic sills are highlighted in red color within the basement unit in (3b). TWTT: Two-way travel time in milliseconds.

The oldest interpreted horizon in this work is equivalent to the top basement in wellbore Kawau-1A (Quartzite), Pukaki-1 (Granite), Pakaha-1 (Granite), and Tara-1 (Gneiss). The granite in the Pukaki-1 well was logged as sandstone and described to consist of transparent to light yellow to orangish pink, medium to coarse-grained, poorly sorted, angular to very angular, disseminated pyrite and mica, chlorite, while the gneiss in the Tara-1 is dominated by dark green, very dense, clear to slightly milky quartz (https://data.nzpam.govt.nz/). On seismic profiles, the basement reflections include chaotic, low to moderate amplitude reflections that are often intersected by deep-seated faults and intrusive rocks (Fig. 3).

**Figure 4:**
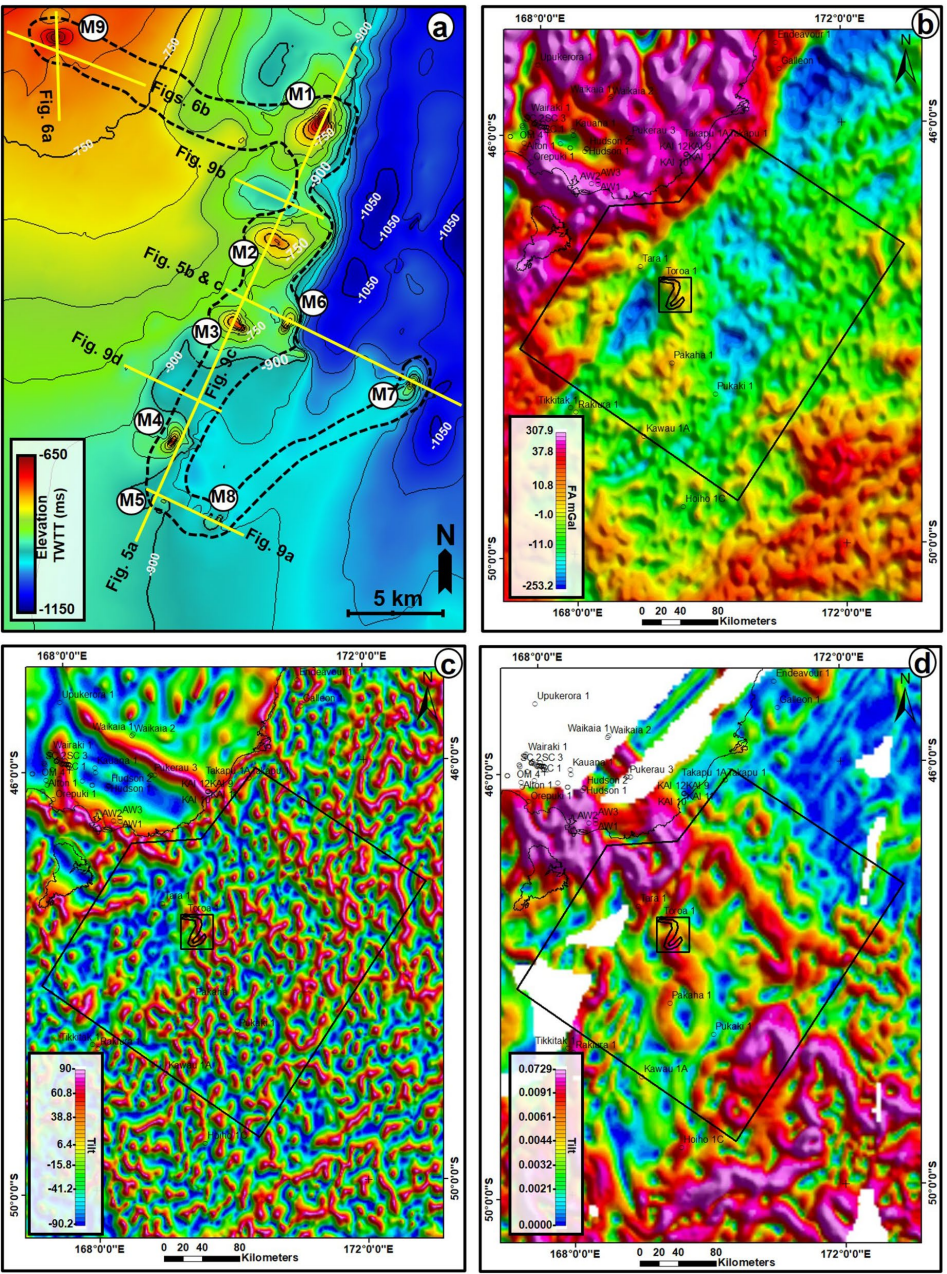
(**a**) TWTT structural map of the seafloor showing the location of the nine mounds (M1-M9) analyzed in this study. Contour spacing for the map is 30 ms TWTT. TWTT: Two-way travel time in milliseconds. The location of this map is shown in Fig. 1b. The black dashed polygon highlights the stretch of the seafloor mounds, which covers an area of about 186 sq. km. (**b**) Free Air (FA) gravity map over the study area and anomalies shown in (**c**) Tilt filter of the Bouguer corrected Satellite gravity data (**d**) Modulus (MS) of Reduced To Pole Magnetics with a 20 km Low Pass filter. (**b**)–(**c**) were reprojected in the NZGD2000, NZTM coordinate system (Data source: www.nzpam.govt.nz).

**Figure 5:**
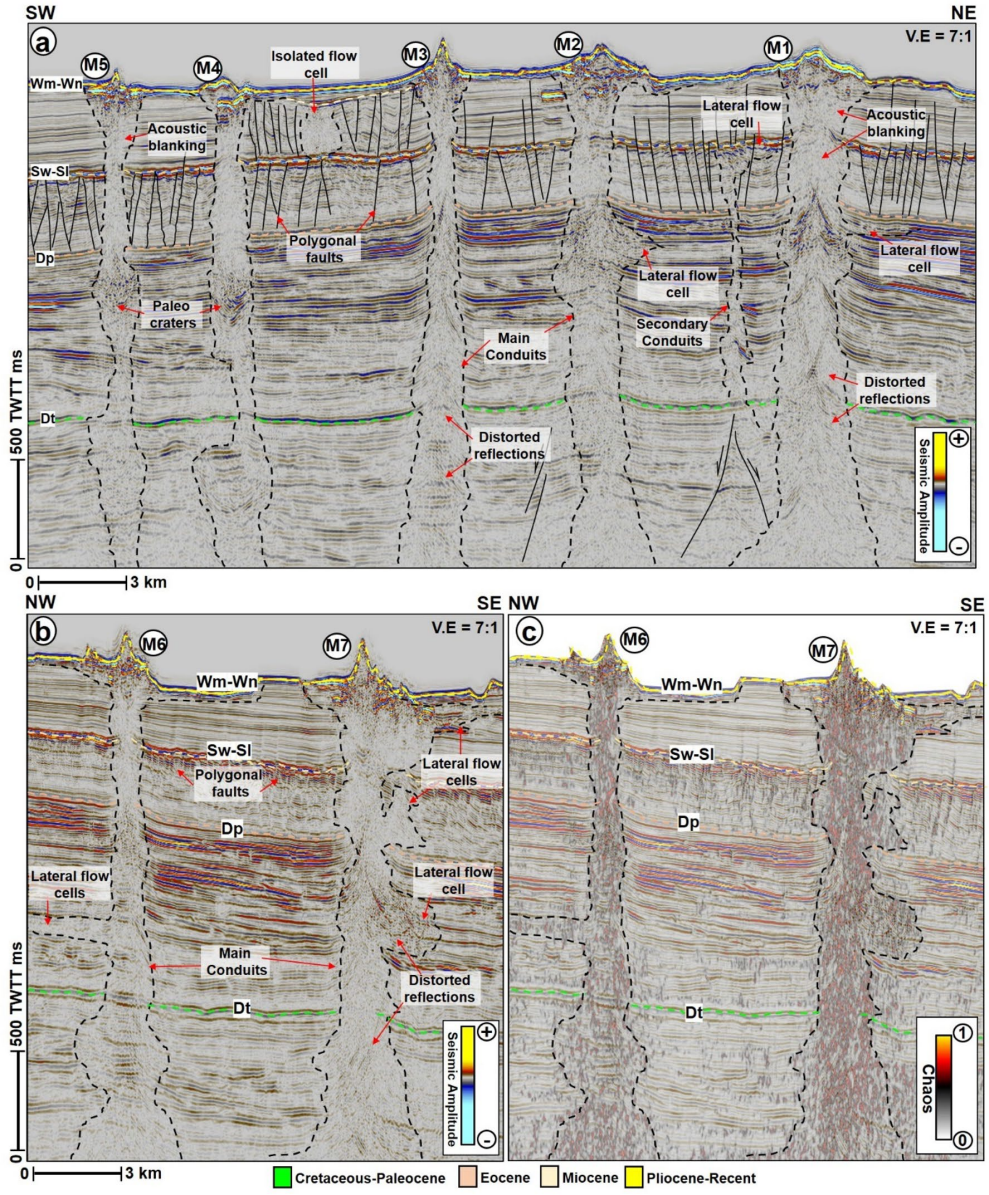
SW-NE seismic profile across mounds M1 to M5. The mounds are connected to the basement by vertical zones of distorted seismic signal interpreted as fluid escape pipes. Average height of the pipes is about 4500 ms TWTT. (**b**) and (**c**) NW–SE seismic profiles showing vertical conduits associated with mounds M6 and M7. In (**c**), the chaos seismic profile is overlain by the amplitude sections to correctly map the outline of the pipes. A striking feature of these pipes is that they are connected in their middle parts to lateral zones of chaotic reflections, interpreted as flow cells. *TWTT: Two-way travel time in milliseconds. The uninterpreted seismic sections are provided in the supplementary document*.

The Cretaceous sediments (Mh and to the basement) are mostly part of the Hoiho Group and are syn-rift sequences (Fig. 3). Accordingly, most of the sediments at the base of the sequence are typified by chaotic, faulted, low frequency, low to moderate amplitude reflections. Above the top of the Cretaceous sequence, the reflections grade into high amplitude and continuous reflections, suggesting a transition from the Hoiho Group and Kawau Sandstone to the overlying Palaeocene Taratu Sandstone and Wickcliffe Formation (Figs. 3, 4, 5). In the western part of the study area, the Cretaceous to Palaeocene strata (Mh and Dt) generally thin and onlap onto the basement (Fig. 3). Moreover, the sequence above Dt are the Eocene strata, which are dominantly represented by high amplitude, high frequency, and continuous reflections (Figs. 3, 4, 5). These high amplitude reflections are interpreted as shale of the Laing Formation (Fig. 2). The chaotic and discontinuous low amplitude reflections at the base of the Eocene sequence are correlated to the Laing Formation Coastal sand facies (Fig. 2).

The Neogene strata are markedly affected by two sets of polygonal fault systems, first at the Miocene level and towards the base of the Pliocene to Recent sediments (Figs. 3 and 5). These polygonal fault systems are especially predominant and appear close to vertical zones of distorted seismic reflections that are associated with the seafloor mounds described below (Figs. 5 and 7). Like in many sedimentary basins^40^, polygonal fault systems in the GSB are layer-bounded normal faults with small offsets but occasionally are linked by few normal faults e.g., Figs. 4 and 5. Apart from the polygonal fault systems, the Miocene strata are represented by high frequency, alternating low and medium to high amplitude reflections. The Miocene strata from Sw-SI to Dp are correlated to the Laing Formation Shelfal Facies in the Tara-1 wellbore (Figs. 2 and 3). Contrastingly, the overlying Pliocene-Recent unit includes alternating high and medium continuous reflections interpreted as hemipelagic sediments of the Penrod Formation. In the western part of the study area, the Neogene sequence is capped by sigmoidal reflections suggestive of predominant deltaic deposition from the west (Fig. 3).

### Seafloor mounds, fluid-escape pipes, and flow cells

**Figure 6:**
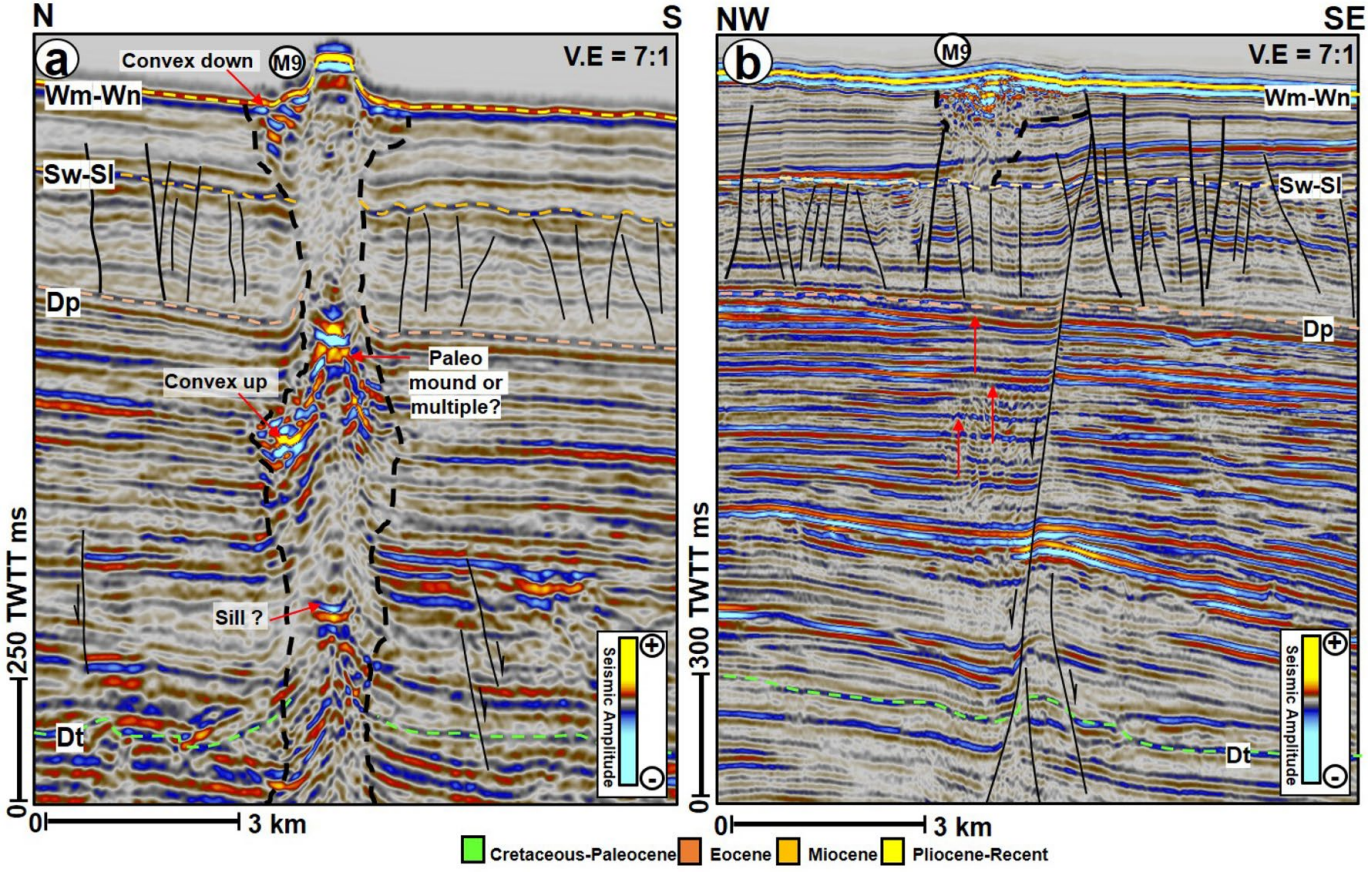
(**a**) N-S seismic profile through mound, M9 showing the vertical succession of mounds within the underlying escape pipe and interaction with magmatic sill at depth and (**b**) NW–SE seismic profile showing the connection between M9, polygonal faults and a deep-seated normal fault. *TWTT: Two-way travel time in milliseconds. The uninterpreted seismic sections are provided in the supplementary document*.

**Figure 7:**
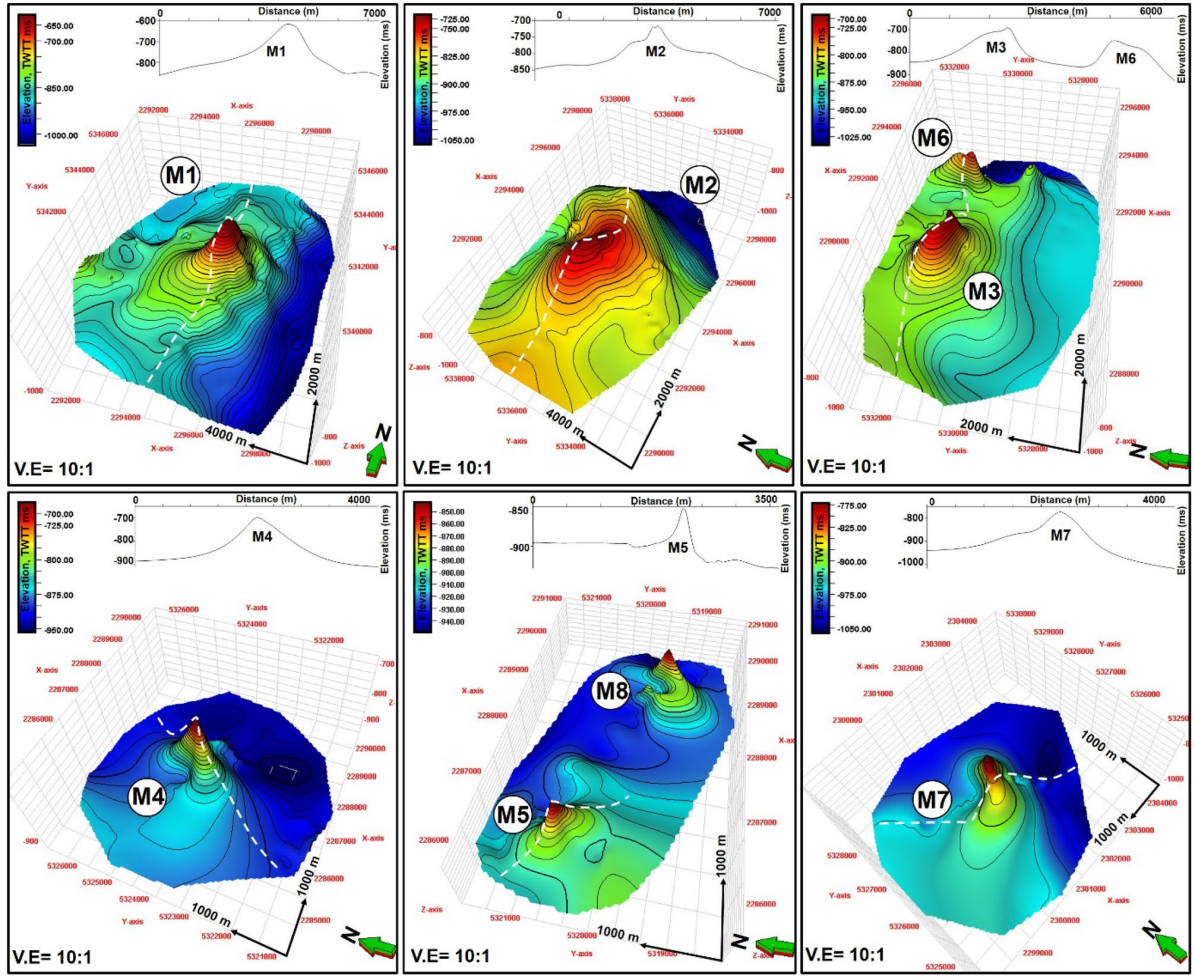
3D map view and cross sections across mounds M1 to M8. The white line across the maps show the location of the cross sections displayed at the tops of each map. *Contour spacing for the structural maps is 5 ms TWTT.* TWTT: Two-way travel time in milliseconds.

**Figure 8:**
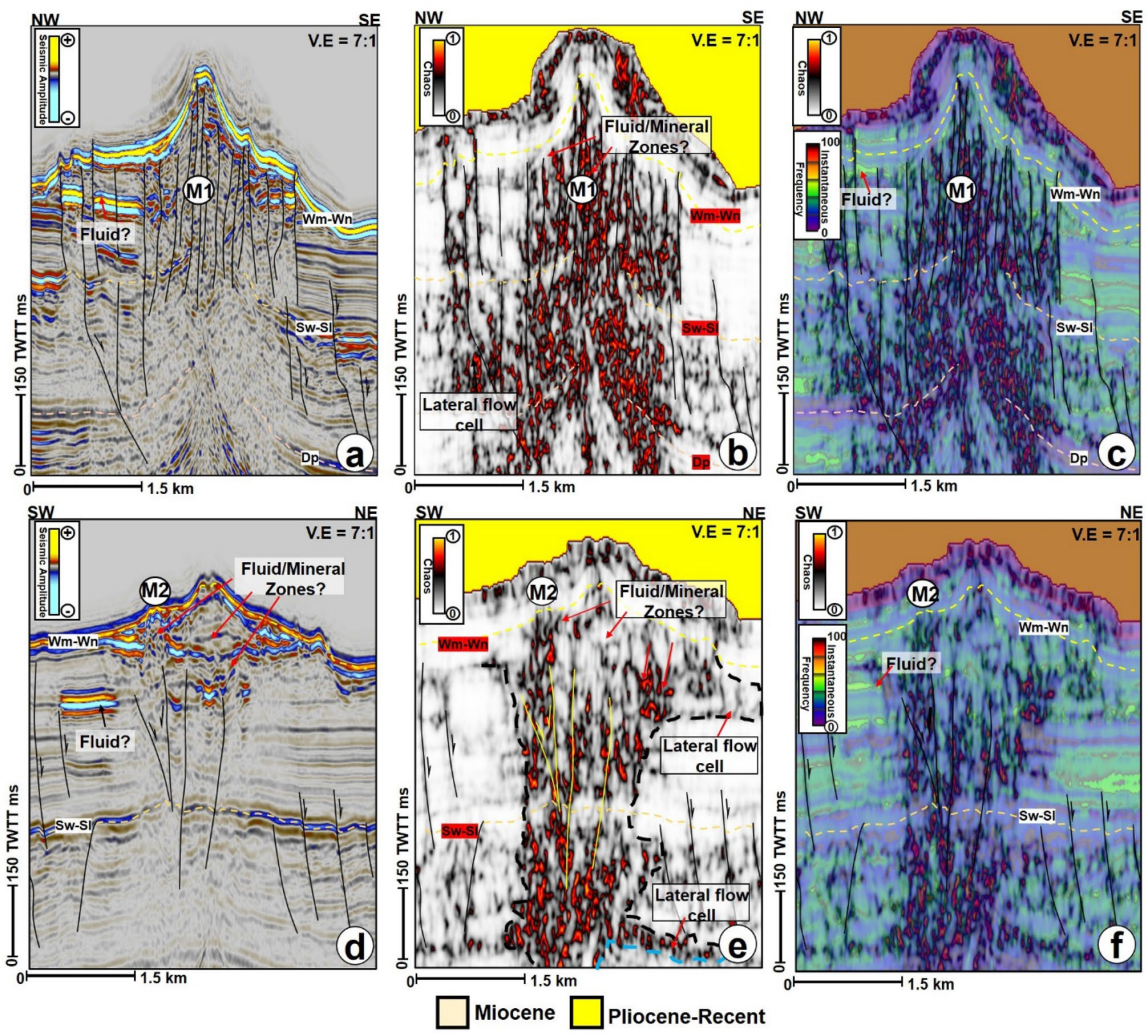
Internal architectures of seafloor mounds (**a**)–(**c**) M1 and (**d**)–(**f**) M2 as revealed by the amplitude, chaos and instantaneous + chaos profiles. M1 and M2 have cone-shaped and plateau-like zeniths, respectively and are intersected by different faults within the Pliocene-Recent strata. *Note: Profiles in* (**c**) and (**f**) *are chaos profiles overlain by instantaneous frequency profiles.* TWTT: Two-way travel time in milliseconds. *The uninterpreted seismic sections are provided in the supplementary document*.

**Figure 9:**
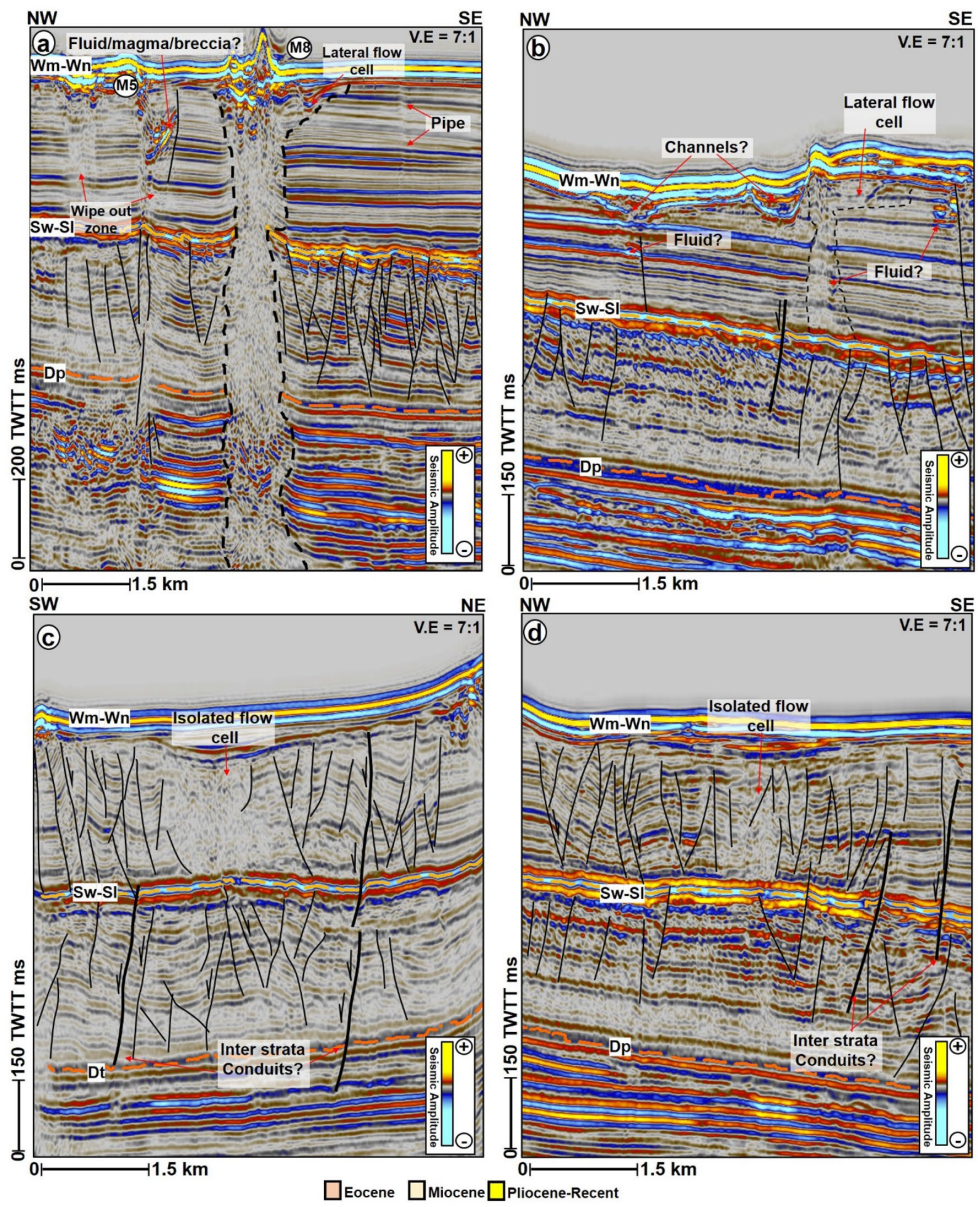
Examples of both vertical and lateral flow cells. (**a**) NW–SE seismic profile showing the interaction of M5 and M8 with other high amplitude anomalies within the Pliocene-Recent strata. In addition, wipe out zones associated with M5 signifying the passage of fluids in the subsurface. (**b**) An example of a lateral flow cell at the northernmost section of M2. Here the link between the Miocene polygonal fault system and the overlying mounds is shown by normal faults connecting both the Miocene and Pliocene-recent strata. (**c**) Along-strike and (**d**) along-dip view of the isolated flow cell shown in Fig. 4. *TWTT: Two-way travel time in milliseconds. The uninterpreted seismic sections are provided in the supplementary document*.

Nine seafloor mounds (M1–M9) were identified in the study area (Figs. 4a, 5, 6, 7, 8, 9). These mounds are part of the Neogene sequence and have cone-shaped upper parts, except M2 that is nearly plateau-like in map view (Figs. 5, 6, 7). Heights of the seafloor mounds can reach up to ca. 137 m (M7), with the shortest mound being M5 (ca. 61 m tall). The lengths of their long axes and areas are 1.1 km to 5.1 km and 0.69 km^2^ to 16.99 km^2^, respectively (Table 1). Most of the mounds are dominantly oriented in the northeast-southwest (NE–SW) direction (Figs. 4 and 8), with their flanks having a maximum angle of dip between 19^0^ to 38^0^ (Table 1). On seismic profiles, the mounds have tops that are characterised by high amplitude reflections. They also have crater- or depression-like bases, comprising a mixture of different amplitude reflections (Figs. 5 and 6). The mounds are delimited in a NW–SE stretch covering an area of about 186 sq. km on the seafloor map (Fig. 4a). On both the Free Air (FA) gravity and Tilt filter of the Bouguer maps, this stretch corresponds with areas of positive gravity anomalies (Fig. 4b and c) while the area also coincide with areas of positive magnetic anomalies on the Modulus of Reduced To Pole (RTP) magnetic map (Fig. 4d).

**Table 1:** Morphometric data for the nine interpreted mounds.

Mound	Shape of mound	Strike	Maximum flank dips	Height of	Height of	Long axis of mound	Short	Area of mound	Pipe
							axis of mound		
		0°	0°	Mound	Mound m	(km)	(km)	(km^2^)	Height
				(TWTT)					(m)
					Velocity of 1400 m/s				Velocity of 2670 m/s
M1	Cone	37	32	153	107.1	4.97	3.42	16.99	5157.06
M2	Flat	89	19	140	98	5.09	3.28	16.70	4355.28
M3	Cone	117	38	159	111.3	3.04	2.06	6.26	4407.72
M4	Cone	47	36	94	65.8	2.43	1.25	3.04	3877.80
M5	Cone	144	28	87	60.9	1.05	0.66	0.69	4128.96
M6	Cone	61	31	158	110.6	1.76	1.06	1.87	5210.88
M7	Cone	57	38	196	137.2	2.31	0.99	2.29	5426.16
M8	Cone	53	30	124	86.8	1.3	0.96	1.25	5354.40
M9	Cone	89	29	127	89	2.42	1.22	2.95	4346.40
Average		77	31	138	96	2.71	1.66	5.78	4696.07
Min		37	19	87	60.9	1.05	0.66	0.69	3877.80
Max		144	38	196	137.2	5.09	3.42	16.99	5426.16

Furthermore, internal configurations of the mounds on seismic profiles include faulted areas comprising low and high amplitude reflections and highly chaotic reflections with moderate to high frequencies (Fig. 8a–c). These faulted segments are interpreted as either discrete mineral domains or fluid flow cells within the mounds (M1 in Fig. 8a–c). A continuous reflection can also be present internally within the mounds, separating the cone at the top from the crater at the base of the mounds (e.g., M2 in Fig. 8d–f). Such internal reflection can be interpreted as evidence for plugging and compaction of the depressions prior to the formation of the mounds^41,42^. Additionally, high amplitude reflections within the underlying craters sometimes are disseminated in nature, suggesting disparate compositional-lithological variation of the craters (see M2, M5, M7, and M8 in Figs. 5 and 8a). Besides their top, basal, and internal configurations, mounds in the study area also show close interactions with other isolated high amplitude anomalies (Figs. 5, 6, 8). Some of these anomalies are soft kick reflections with opposite polarity to the seafloor reflection (Fig. 8). Hence, they are interpreted as fluid accumulations in the subsurface (e.g., M1 and M2 in Figs. 5a and 8). On the contrary, the other type of high amplitude anomaly close to M5 is interpreted as a magmatic sill based on its hard kick character (Fig. 9a). All the isolated high amplitude anomalies associated with the mounds are frequently flagged along the hanging wall sections of normal faults (Figs. 6 and 7), an indication that faults were active fluid conduits in Pliocene to Recent times.

A striking characteristic of all seafloor mounds in the study area is that they are located above sub-vertical zones of low amplitude, washed out reflectivity, and distorted reflections. These zones are interpreted as gigantic fluid-escape pipes^43,44^ (Figs. 3b, 5, 6, 9a). Pipes beneath the mounds are generally upward narrowing in seismic sections, except for the ones beneath M5 and M7 that are widening towards the top (Fig. 5). All the pipes are also rooted either within the basement (Fig. 3) or Cretaceous units (Figs. 5, 6). Their height ranges from ca. 3.9 to 5.4 km (Figs. 5, 6, 9a, Table 1). A common attribute of the pipes is that they frequently exhibit high amplitude reflection packages near the top Eocene units, forming paleo craters (Figs. 5, 9a) or a paleo mound at this level (Figs. 5, 6a). These high amplitude reflection packages contrast sharply with the background low amplitude and distorted reflections prevalent within all the pipes.

In addition to the interpreted paleo craters and mounds, the pipes that are not entirely upward narrowing in shape are occasionally affected by local excursions into isolated lateral zones of low amplitude and chaotic reflections interpreted here as lateral flow cells (e.g., M1, M2, M6, and M7). These lateral flow cells are not seafloor multiples of the mounds as they are disparate in reflectivity, morphology, and size from their overlying mounds. In this study, the lateral flow cells can be present at all the stratigraphic levels, including close to the seafloor reflections but are predominant within the Eocene strata (Figs. 5, 8b,e, 9a,b). A compelling isolated flow cell (Figs. 5a, 9c, d) that is classically composed of low amplitude and contorted reflections within the younger polygonal fault system is observed between the pipes below M3 and M4. This feature along dip resembles a collapsed structure within the Pliocene-Recent polygonal fault system (Figs. 9c,d). Polygonal faults and a few normal faults are regularly seen associated with the lateral flow cells close to the seabed (Figs. 5 and 6b). In the case of M9, the polygonal faults are intersected by a deep-seated fault that extends into the Cretaceous interval (Fig. 6b). Aside from the lateral flow cells, some of the pipes also locally interlink with other secondary pipes to give the outlook of bifurcated pipes within the Cretaceous to the Paleogene strata (Figs. 5a and 9a).

### Volcanoes and intrusive rocks

**Figure 10:**
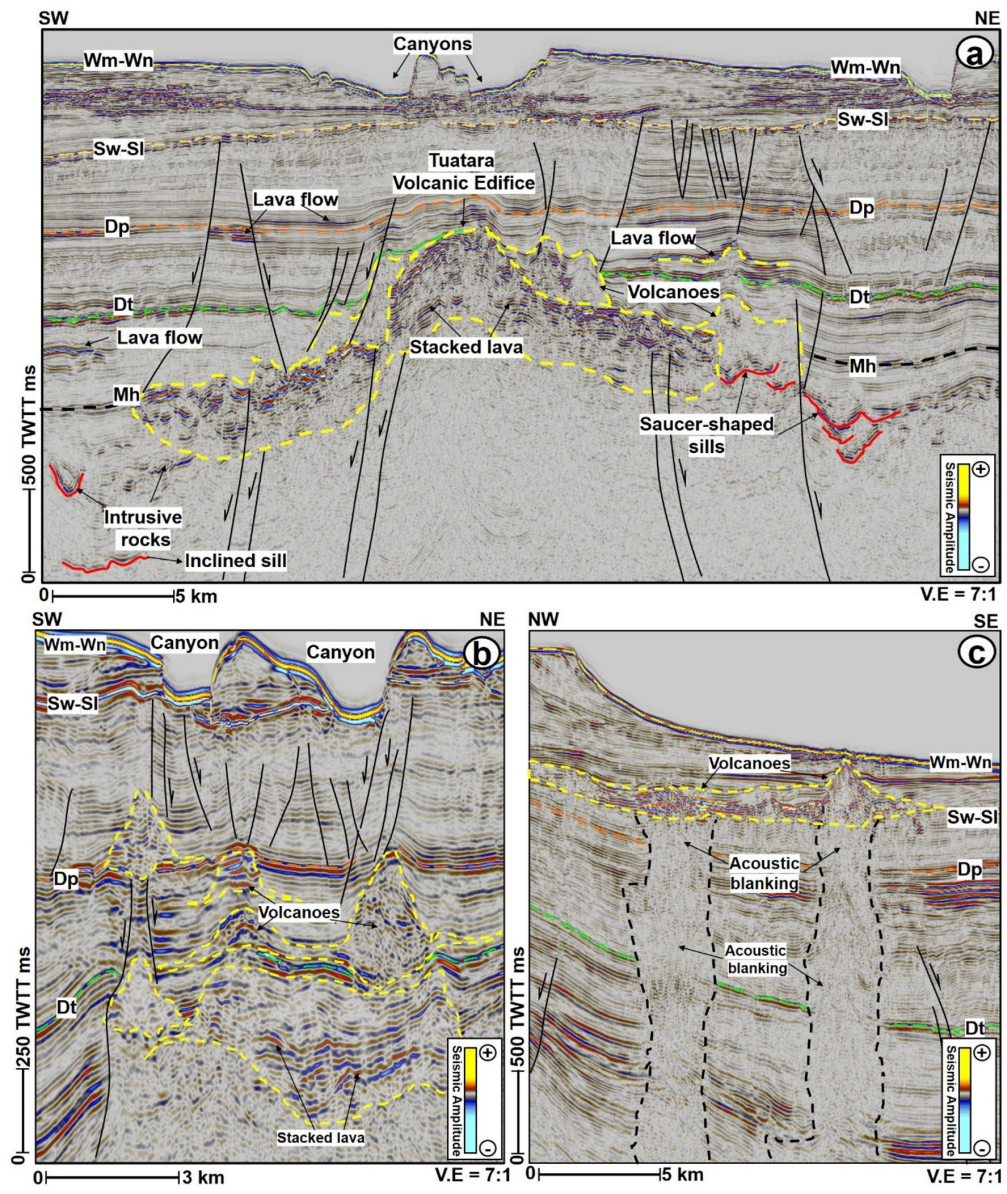
(**a**) Interpreted SW-NE seismic section showing the architectural outlook of the Tuatara Volcanic Field (TVF) of^32^. The volcanoes here are mostly interpreted within the Paleocene strata, especially on the eastern flank of the TVF. Seismic sections showing other volcanoes identified in the study area. These include (**b**) those that are vertically and laterally stacked within the Eocene strata and (**d**); those seen emplaced within the Miocene to Pleistocene strata. These volcanoes are also commonly found with lava flows and intrusive rocks. *The uninterpreted seismic sections are provided in the supplementary document*.

Volcanoes in the study area include an edifice in the NE part of the seismic surveys (Figs. 1b and 10a). This edifice is interpreted as the domal structure of the Tuatara Volcanic Field^32^. On its flanks, several lateral and vertical stacks of high amplitude reflections are interpreted as associated lava flows (the horizontal ones) or intrusive rocks (the near saucer-shaped reflections). Volcanoes interpreted around the Tuatara Volcanic edifice are typically cone-shaped and on the SW of the edifice are strongly intersected by normal faults (Fig. 10a). Importantly, the main cone-shaped volcanoes adjacent to the edifice are buried within the Palaeocene strata, while the lava flows and smaller volcanoes are interpreted within the Eocene interval (Fig. 10a). A classic association of vertical and lateral stack of cone-shaped volcanoes is interpreted north of the Tuatara Volcanic Field (Fig. 10b). These buried volcanoes are present at three different stratigraphic intervals i.e., Palaeocene, Eocene, and Miocene (Fig. 10b), signifying a prolonged history of volcanism in the study area from the Palaeocene to Miocene. Volcanoes associated with the Miocene and Pleistocene units are also mapped directly beneath the present-day seabed, close to the seafloor mounds (Figs. 1b, and 10c). Additionally, intrusive rocks in the GSB (Figs. 3 and 10) often intersect the basement unit and sometimes are present on both the footwall and hanging wall sections of some of the deep-seated faults (Fig. 3a,b). Those associated with the volcanoes are found in two main domains, defined as the western domain comprising patches of intrusive fields and an eastern domain composed of two isolated intrusive fields in Fig. 1b. These two domains run essentially NE-SW with the western domain extending south and subjacent to the mapped seafloor mounds (Fig. 1b). Importantly, one of the intrusive fields is mapped close to the Toroa wellbore. This may correspond to the Toroa volcanic field interpreted by^36^. Apart from these two domains, it is also possible that there are more intrusive rocks buried within the Palaeocene and Cretaceous units. Perhaps, these unmapped intrusive rocks are too thin and at sub-seismic resolution^42^.

## Discussion

### Concatenation of fluid-flow cells and plumbing system of the area

**Figure 11:**
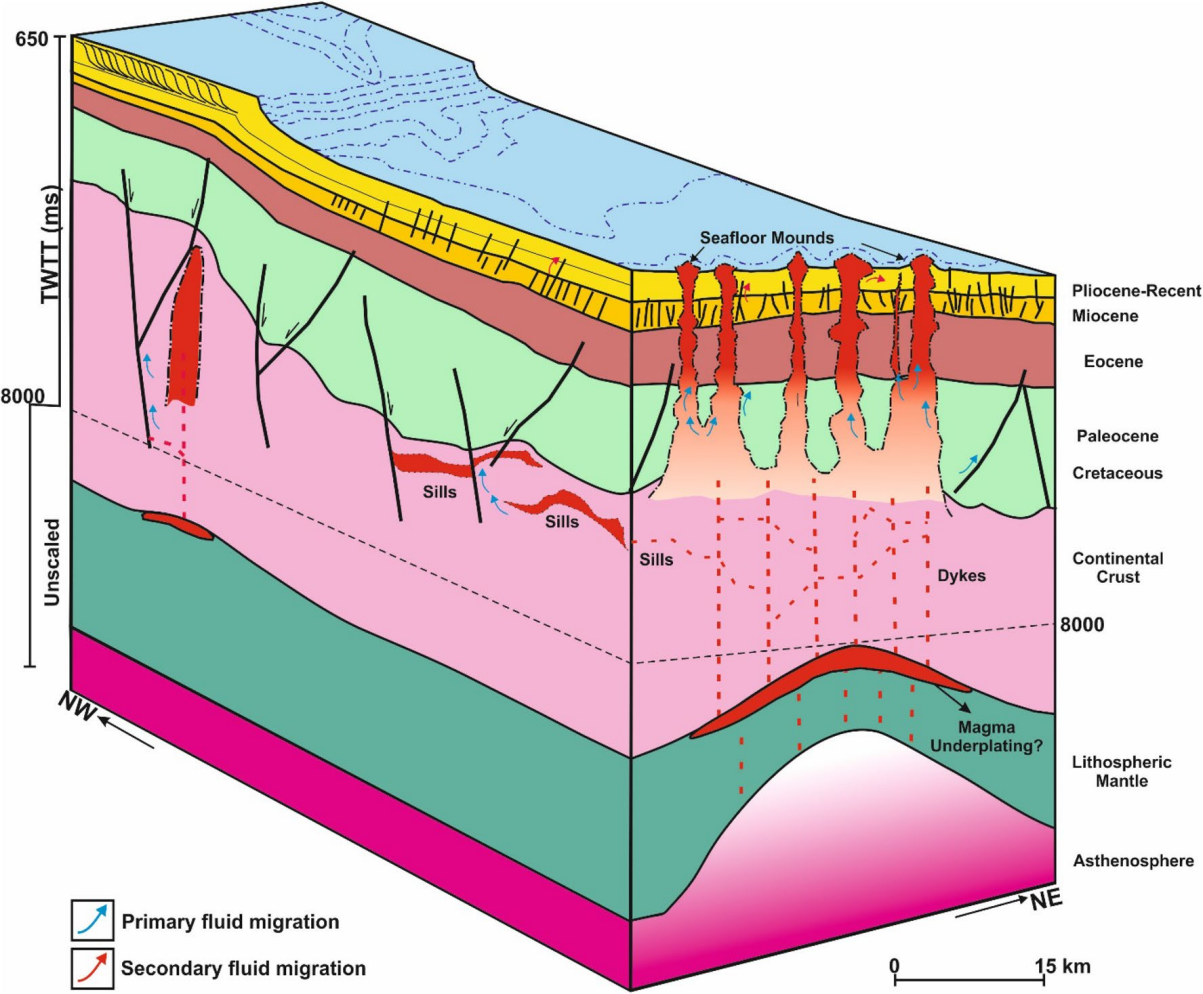
Conceptual diagram showing the link between the seafloor mounds, their vertical conduits and the fluid pumping system in the study area. Figure inspired by ^97^ and ^12^. TWTT: Two-way travel time in milli-seconds.

A conceptual model describing the fluid sources and plumbing system of the study area is provided in Fig. 11. Fluid flow in the study area is primarily associated with vertical migration of magma from the basement through the Cretaceous up till the Pliocene-Recent sequence (Fig. 3). Vertical fluid flow in the study area is protracted in nature and possibly occurred in at least two phases. A first phase involving the formation of massive paleo craters that are common near the top of the Eocene level (Dp) and a second phase when the pipes were reutilised for upward migration of magmatic fluids (Fig. 5). The craters at the base of the present-day seafloor mounds were likely formed during a secondary phase of vertical fluid migration. In addition to the multiple cross-stratal migration of magmatic fluid through the pipes, fluid mixing as a result of lateral or along-bedding fluid migration from different source rocks into the pipes and overburden is also likely in the study area (Fig. 11).

Intrusive rocks directly beneath the pipe of M1 for example suggest a causative link between magmatic intrusion and pipe formation (Fig. 3). Such interaction between intrusive rocks, fluid escape pipes, and crater/mound formation has been documented in literature, especially along magma-rich continental margins^43,45^. In the study area, pipes were formed due to the build-up of critical fluid pressures when fluid is released during either metamorphic dehydration or when fluid boils within the host-rock aureoles. Fluid overpressures related to metamorphic dehydration reactions can cause gases, such as CO_2_, SO_2_, and halocarbons to be released, triggering catastrophic blowouts and creating pipes^46,47^. Also, pressure increases due to magma intrusion would lead to boiling of the host rock, followed by degassing of the intruded magma and subsequent fracturing of the overburden, creating vertical conduits, such as the pipes observed here^48,49^. Thus, the pipe-intrusive rocks architecture (Fig. 3) provided marked evidence for the primary source of fluids to be magmatic.

Secondary sources of fluids in the area can include fluids from (a) coal beds and coaly mudstones within the Cretaceous and Paleogene sequences, and (b) foraminiferal oozes from the Pliocene-Recent sequence (Fig. 9). The lateral flow cells intersecting the pipes are proofs for along bedding migration of fluids potentially from source rocks at the Paleogene and Cretaceous levels (Fig. 5). Fluid flow cells in this work define geologic compartments or areas where fluid is locked up due to fault intersection or changes in stratigraphic facies. More so, the mid-Cretaceous coaly sediments of the Hoiho Group have been documented as the most likely sources of gas condensate and oil shows encountered in the Kawau-1A wellbore^27^. Oil and gas shows are also recorded in the Tara–1, Toroa–1, and Pakaha–1 with minor methane detected in the Pukaki-1 well^27,38^. At the Paleocene level, the Taratu Formation in Tara-1 is characterized by very high percentages of degraded brown phytoclasts, rare marine algae, and amorphous organic matter portraying a mix of terrestrial and marine kerogen^29^.

The Miocene and Pliocene sequences are further characterized by polygonal fault systems, which are valid proxies for relict episodes of fluid flow in the study area^44,50^. Polygonal faults are caused by layer-bound contraction-driven shear failure during the early stages of sediment compaction and dewatering in subsiding sedimentary basins^40,51^. Polygonal fault systems are common mostly in basins that are free of lateral tectonic forces^52^. In the study area, the polygonal fault systems are proposed as high drainage interval or the main conduits supplying fluids into the Pliocene-Recent lateral flow cells (e.g., M2 in Fig. 5a). Normal faults are also observed to recurrently connect the two sets of polygonal faults observed within the Pliocene-Recent interval, thus, aiding vertical migration of fluids into the overburden and to the lateral flow cells, respectively (Figs. 5a and 9b–d). The Pliocene-Recent strata are also rich in foraminiferal oozes with chalk and chert^30^, which potentially are sources of connate fluids or gases within the lateral flow cells. Hence, a prolonged history of mainly vertical migration of magmatic fluids, intermittently overlapping with multiple lateral fluid migration from coaly and foraminifer-bearing source rocks is prescribed for the study area.

### On the origin and types of seafloor mounds in the study area

The architecture of the seafloor mounds excludes them from being described as mud volcanoes, gas hydrate pingoes, and methane-derived carbonate reefs. Although compression-related mud diapirism could have occurred during regional fault reactivation and uplift in the Oligocene-Quaternary^53^ and may be able to force deeply buried materials to the seafloor to form mounds^54,55^. However, such geological features are not compatible with the seismic character observed for the mounds in the study area. Mud volcanoes are usually characterised by high amplitude top and base reflections, with their internal seismic facies composed of chaotic to discontinuous, low frequency and low amplitude reflections, with some continuous stratification and amplitude anomalies. Hence, the internal compositions of mud volcanoes are described as “acoustically transparent”^54,55^. On the contrary, the mounds here (M1-M9) are composed of heterogenous mixture of faulted, high amplitude, moderate to high frequency reflections, are not acoustically transparent and are dominantly cone-shaped. Their conduits also lie above volcanic and basement rocks (Fig. 3), a behaviour that is unlike the archetypical zone of withdrawal below mud volcanoes^8^. Mud volcanism can hardly explain the conical shape of the mounds and their relationships with underlying volcanic sill intrusions.

Also, the seafloor mounds resemble gas hydrates pingoes in their form^2^, but no shallow gas hydrate related Bottom-Simulating Reflectors (BSRs) exist in the study area. Occurrences of gas hydrates have been documented in other New Zealand basins such as the Taranaki Basin, Fiordland and Hikurangi Margins^56–58^. For instance, seafloor domes in the Opouawe Bank, Hikurangi Margin are linked to gas migration and gas hydrate systems^57^. On the contrary, there is no record of gas hydrate in the Great South Basin yet as no geophysical evidence to suggest its occurrence has been reported. In fact^59^, modelled the stability of gas hydrate in the study area using a geothermal gradient of 39 °C/km and seabed temperature of 5 °C and concluded that the BSR in the Great South Basin is too deep to form present-day gas hydrate. Heat flows in the Great South Basin are also quite high (ranging from 87.2 to 163.8 mW/m^2^) and are geologically unfavourable for the formation of gas hydrate. These high heat flows are attributable to the presence of young volcanism and intrusions located above the basement unit^38,60^.

Although, the seafloor mounds show some similarities with methane-derived carbonate mounds such as high reflectivity and by being underlain by poorly imaged zones^61,62^. However, their geometries, large sizes, lack of internal layering, and sharp boundaries signify that they are unlikely to have evolved mainly as methane-derived carbonates on the seafloor. Notwithstanding, we do not rule out the possibility that the mounds may contain some accumulations of authigenic carbonates or shells of endemic marine organisms, that could have relied on fluids plumbed via the underlying conduits and vented on the seafloor. Consequently, the seafloor mounds here are interpreted to be volcanic in origin, with fluids being derived and plumbed mainly via hydrothermal processes that post-dated Cenozoic intraplate volcanism in the GSB. Important volcanic edifices in the study area include the Tuatara volcanoes which were emplaced over 40 Myr^32^ in the northern part of the study area and Pleistocene volcanoes^36^ which were emplaced in the area south of the Toroa-1 well, close to the mapped seafloor mounds (Fig. 1). Hence, hydrothermal processes associated with these volcanic edifices could possibly have occurred several years (up to 50 Ma) after the emplacement of the volcanoes (see^63–65^), especially if the magma migration pathways have prolonged residence times^66^. A volcanic origin for these mounds is reinforced by the presence of Palaeocene-Pleistocene buried volcanoes directly beneath the seabed (Fig. 10c), the geophysical expressions and sizes of the seafloor mounds (Fig. 4a–d), connection between the mounds and buried sill intrusions (Fig. 3) and by the presence of several isolated Cenozoic intrusive rocks close to the mapped seafloor mounds (Fig. 1b).

### Implications for seafloor mineral deposits in the GSB

The structural make-up of the seafloor mounds and by extension their associated fluid escape pipes plus intrusive rocks fit the generic model developed for metalliferous mineral deposits such as the giant (150 Mt) Sullivan Pb–Zn-Ag deposit in the Mesoproterozoic Belt-Purcell Basin, south-eastern British Columbia. Sullivan-type mineral deposits can evolve in four main stages^12,67^ through (a) emplacement of intrusive rocks and development of a large mud volcano complex; (b) continued sill-related heating and formation of epigenetic tourmalinite through the rise of a low-salinity, condensed vapor derived from sill-sediment interactions; (c) formation of Pb–Zn-Ag sulphide deposits related to release and transport of a buoyant, high-salinity brine and (d) Sn-rich cassiterite mineralization in which a new hydrothermal system was generated by the emplacement of younger mafic sills and dikes in the shallow subsurface. Similarly, the multiple episodes of volcanic activities which characterized the GSB permitted the emplacement of intrusive volcanic bodies, leading to the development of the km-scale mounds on the seafloor. These mounds were most likely formed as hydrothermal activities drove upward plumbing of mineralized fluids via the subsurface pipes, fostering the deposition of massive sulphides or ore-grade mineral deposits on the seafloor.

Although, there are no ground truth data like seafloor observations, cuttings, or well penetrations through the mounds studied here, the strong reflection at the top of the mounds, their shapes, characteristics, variable internal reflections and associated architectural elements (Figs. 3, 4, 5, 6, 7, 8, 9, 10) further depict them as potential seafloor massive sulphide or mineral deposits. More so, the unique presence of craters at their bases and connection of their pipes to intrusive rocks at depth support the idea that the pipes could have served as conduits to convey mineralized fluids leading to the accumulation of massive sulphides on the seafloor. Seafloor massive sulphides have significantly higher acoustic impedances than silicate rocks, and as such can be directly interpreted from seismic reflection data provided the deposits meet the geometric constraints required for seismic detection^68^. Additionally, the mounds here are also associated with strong magnetic and gravity anomalies due to their strong magnetic susceptibility and high densities, which could point to the occurrence of mineral deposits^69–71^, while their geometries and architectural elements are akin to the archetypical seafloor mineral mounds reported in many settings^72^ and hydrothermal vent complexes observed along many magma-rich margins^64,73,74^. Therefore, the potential for seafloor mineral/metal deposits should be investigated in the GSB and other basins with similar geological setting.

## Materials and methods

### Seismic and well data

The seismic data used in this study are multiple 2D seismic profiles acquired during the OMV08 and DUN06 surveys (Fig. 1b). Detailed acquisition and processing parameters for both surveys are provided in reports PR3392 and PR3450, respectively (https://data.nzpam.govt.nz/). The OMV08 seismic survey consists of 120 seismic lines covering about 16,010 km, while the DUN06 survey consists of 23 high-quality seismic profiles covering 3110 km in the northern part of the GSB. Both surveys were acquired with 480 channels, 6000 m streamers, a nominal fold of 120, recording length of 8000 ms Two Way Travel-Time (TWTT), and a vertical sampling interval of 2 ms giving a maximum theoretical frequency of 250 Hz (Nyquist frequency). The DUN06 survey is important because it ties to five of the eight wells drilled in the GSB i.e., the Toroa-1, Kawau-1A, Tara-1, Takapu-1A, and the Pukaki-1 wellbores (Fig. 1b). For display purposes, all the seismic profiles are zero-phase processed and displayed with Society of Exploration Geophysicists (SEG) normal polarity, whereby a downward increase in acoustic impedance is a positive reflection (peak/red reflection at the seabed) and a downward decrease in acoustic impedance is a negative reflection (trough/blue reflection). Other positive reflections with similar polarity as the seabed are considered as hard kicks or lithology related features while soft kicks with opposite polarity to the seabed reflection are interpreted as fluid related features. Furthermore, well tops and biostratigraphic data from the Toroa-1, Kawau-1A, Tara-1, and the Pukaki-1 wellbore were used to constrain the ages of the interpreted horizons and the lithology of their bounding strata. All the wells were terminated within hard (igneous or metamorphic) rocks of different types at TD, except the Toroa-1 well (Fig. 2). The average velocities of the stratigraphic units from the Pakaha-1 wellbore and published works^75^ are 2760 m/s for the Pakaha Group, 2120 m/s for the Rakiura Group, and 1400 m/s for the overlying Penrod Group. Therefore, by adopting a dominant frequency of ca. 50 Hz for the data (typical frequency for seismic data), the limits of vertical resolution, i.e., λ/4^76,77^, are approximately 7–11 m and 11–14 m from the Penrod to Rakiura and Pakaha Groups, respectively. The limit of visibility (λ/30), for the thinnest feature that will be detected in the seismic data are ca. 0.9 m, ca. 1.4 m and ca. 1.8 m for the Penrod, Rakiura and Pakaha Groups.

### NZ SEEBASE Gravity and Magnetic maps

In addition to seismic and well data, magnetic and gravity data from the study area were used to support the interpretation of the seafloor mounds. Both the gravity and magnetic maps were processed and transformed and readily available from the NZ SEEBASE database^78,79^ while the interpretation of the anomalies followed the works of^33^ and^18^. For example, the gravity map includes a FA Gravity Anomaly and the Tilt filter of the Bouguer corrected Satellite gravity maps. The Tilt filter is calculated from the ratio of the first vertical derivative (1VD) and the modulus of horizontal gradients (MS). It is a useful transformation for enhancing both strong and weak anomalies at their centres and marking out edges of broad anomalies. Unlike the 1VD, the amplitudes are greatly condensed to a small range, and therefore the anomalies appear sharper. The magnetic data presented here are reduced to pole in order to place the anomalies vertically above the magnetic source. Importantly, the Modulus (MS) of Reduced To Pole Magnetics with a 20 km Low Pass filter map was used to highlight the magnetic responses of the seafloor mounds. The MS or Horizontal Gradient Magnitude (HGM) is useful for outlining the boundaries of magnetic sources and it is calculated from a pair of orthogonal horizontal derivatives. The resultant field consists of positive values. The peaks of horizontal modulus anomalies indicate the edges of a source body^80^.

### Seismic interpretation and characterization of seafloor mounds

Seismic interpretation involved mapping of five horizons which are correlated to well tops in the Toroa-1 wellbore. These horizons correspond to the top basement reflection, the top of the Cretaceous (Mh)-Palaeocene (Dt), Eocene (Dp), Miocene sequence (Mid Miocene, Sw–SI), and the present-day seabed (the stratigraphic dates for New Zealand is provided in www.gns.cri.nz). For characterisation of the seafloor mounds on seismic profiles, seismic attributes such as chaos and instantaneous frequency were used. The chaos seismic attribute maps the chaotic signal pattern contained within a unit of seismic data and measures the “lack of organization” in the dip and azimuth estimation method. Chaos attributes are useful for mapping gas chimneys and other vertical structures related to fluid migration pathways and intrusions in the subsurface^81^. In this study, the chaos attribute was used for characterising the seafloor mounds and their associated conduits. Instantaneous frequency (f_ins_) is defined as the rate of change of phase over time (a derivative of the instantaneous phase). It is calculated by the following equation: f_ins_(t) = d(θ_ins_(t))/dt. Where θ_ins_(t) is instantaneous phase and t is time^76^. Instantaneous frequency is useful for reservoir facies characterisation and used in this work to probe the internal architectures of the seafloor mounds by overlaying the instantaneous frequency profiles over the chaos profiles. Both seismic attributes were used carefully as some of the features presented on them might be geophysical noise or artefacts. Hence, their applications were corroborated by regular validation with the amplitude seismic sections. Furthermore, intrusive rocks within the basement and Paleogene rocks are interpreted as magmatic sills based on their amplitude character, similar polarity as the seabed reflection, geometries, and lateral continuity. These rocks have high amplitude relative to the chaotic and low amplitude reflections of the basement rocks. Thus, reflecting that they have higher densities and seismic velocities than their host-rock strata^82^. More so, intrusive rocks have a remarkable seismic stratigraphic expression on seismic profiles and are often characterised by local transgression across stratigraphic levels, restricted lateral continuity, and or/crosscutting relationship with the host-rock strata^83^. Intrusive rocks can also exploit conduits such as faults to migrate from deeper to shallow stratigraphic levels^84^.

### Caveats and confidence in the seismic interpretation of the mounds, pipes, fluid anomalies and intrusive rocks

The seafloor mounds, pipes, magmatic sills (intrusive rocks) and volcanoes interpreted in this work are not drilled or sampled. Hence, their interpretations are based on the knowledge of the general geology of the area^85^, history of volcanism and magmatism in southern South Island of New Zealand^33–35^, their marked physical properties, acoustic contrasts relative to their host rocks^69–71^ and seismic expressions as observed from other margins^86,87^. Additionally, the convex-upwards morphology of some of the paleo craters and mounds at the Eocene levels are cautiously interpreted considering that some of them may be geophysical velocity ‘pull-up’ artefacts. This kind of artefacts are habitually associated with pipes, reefs or similar vertical fluid structures and are related to seismic energy travelling through an overlying high-velocity layer^87^. In a seismic partial reprocessing of the pre-stack version of the DUN06 survey^88^, show that similar structures beneath mounds like those interpreted here are likely seismic processing artefacts or seabed multiples. This author also evaluated the mounds in the DUN survey in the context of bioherms, mud volcanoes, volcanic mounds and concluded these features have a magmatic origin. In parallel, the irregular shapes of the pipes, the linkage between some of them and their termination above inferred basement rocks strengthen their interpretation as fluid escape pipes, rather than seismic imaging artefacts caused by poor imaging below the high amplitude seafloor mounds at the top of the pipes^42,89^.

Similarly, the interpretation of the volcanoes in this work is buttressed by the identification of sub-vertical zones of chaotic and distorted reflections (blanking effects) beneath them, which are likely igneous dykes or pipes, forming a vertical plumbing system to individual volcanoes (e.g., Fig. 10c). The distortions in the reflections may signify disruption of sediment layering as the pipes formed. The variable nature of the distortions in the study area, the likely occurrence of artefacts within/around them, and the complexity of imaging their near vertical flanks makes it difficult to quantify the distortions and to interpret them^90^. However, since the width and seismic responses of the distortions (inferred pipes) are different from those of the seafloor mounds above, our interpretation of the lateral flow cells was guided by the lateral protrusion of low amplitude pipe zones into surrounding high amplitude sediments. These protrusions likely indicate lateral fluid incursion or lithology change, and result in the irregular shapes of the pipes (Fig. 5). Besides, the chaos seismic attribute which highlights chaotic textures within seismic datasets such as fluids^91^ aided the interpretation of the pipes and lateral flow cells zones, both of which are marked by high chaos unlike the low chaos response from surrounding layered sediments.

As for intrusive rocks (sills) in the study area, they have been interpreted solely based on their high-amplitude reflections, similar positive polarity reflections as the seabed, lateral discontinuity, and complex shapes which include transgressive, saucer-shaped, or inclined geometries. Similar features have also been interpreted as magmatic sills in other parts of the GSB^75^. Moreover, they show profound lateral continuity and crosscutting relationship with the host-rock strata (^42^). Their high amplitude reflectivity within otherwise low amplitude reflections indicate differences in densities and seismic velocities of intrusive rocks as compared to their surrounding strata^92^. These physical differences result in high acoustic-impedance contrasts at the intrusive–host rock contacts^82^. Therefore, the intrusive-host rock contacts reflect more seismic energy to the surface than the low-impedance boundaries characteristically occurring between sedimentary rocks^93^. Moreover, only a few magmatic sills (intrusive rocks) are identified in the study area. However, there might be more sills than imaged by the seismic data as magmatic sills are often reflected on seismic sections as tuned reflections with tops and bases that cannot be distinguished^82,93^. Hence, their thicknesses are between the limit of vertical resolution, i.e., λ/4 and the limit of detectability, i.e., λ/32^94,95^, making it difficult to distinguish them from real features (Smallwood and Maresh, 2002). This is most problematic in the GSB at deeper depths where resolution is lower, and the geology is dominated by crystalline rocks. Other sills might also have been omitted due to other geophysical problems such as frequency of the data, inadequate velocity models, sill thickness, overburden complexity, interference between the reflections from closely spaced sills, and the style of the host rock^93,96^.

## Supplementary Information

Supplementary Information

## Data Availability

Can be accessed from the New Zealand Government through New Zealand Petroleum and Minerals (www.nzpam.govt.nz).
