# Supplementary material for: Massive seafloor mounds depict potential for seafloor mineral deposits in the Great South Basin (GSB) offshore New Zealand
Source: Sci Rep. 2021 Apr 28;11:9185. doi: 10.1038/s41598-021-88620-x (PMC8080569; doi:10.1038/s41598-021-88620-x)
Supplement: Supplementary file 1 — Supplementary Information [file 41598_2021_88620_MOESM1_ESM.docx]

**Massive seafloor mounds depict potential for seafloor mineral deposits in the Great South Basin (GSB) offshore New Zealand.**

Omosanya Kamaldeen Olakunle^1,2*^, Lawal Muhedeen Ajibola^3^, Muhammad Iqbal H^4^, Yizhaq Makovsky^3^

^1^ Oasisgeokonsult, 7052. Trondheim, Norway.

^2^Formerly at the Department of Geoscience and Petroleum, Norwegian University of Science and Technology. Trondheim, Norway.

^3^Dr Moses Strauss Department of Marine Geosciences, University of Haifa, Israel.

^4^Department of Earth and Environmental Sciences, Bahria University, Islamabad, Pakistan.

**Corresponding author**:

Omosanya, Kamaldeen Olakunle

Email: [kamal.omosanya@oasisgeokonsult.com](about:blank)

ORCID: 0000-0001-8959-2329


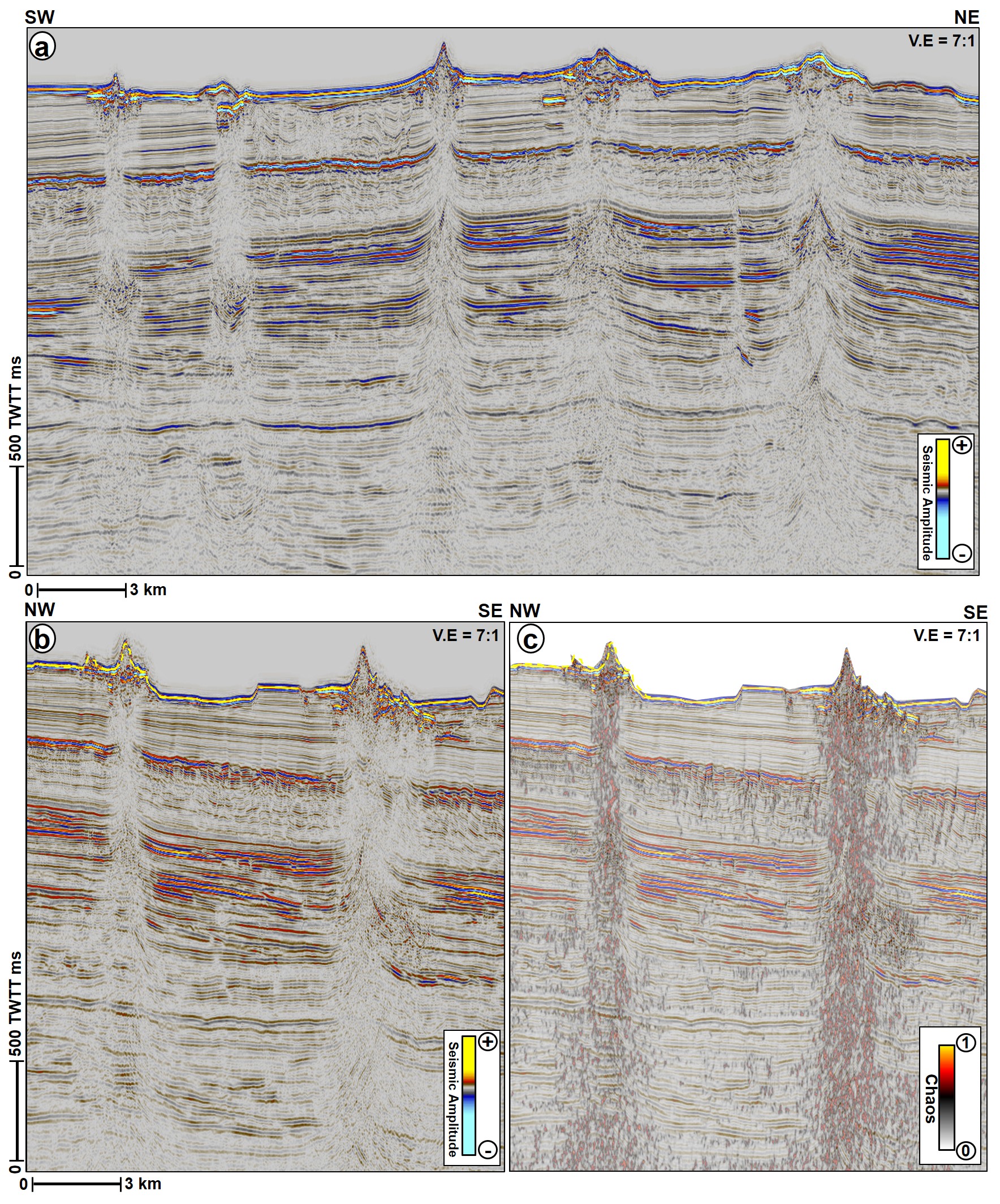


Figure S1: Uninterpreted seismic sections of Figure 5 in the main text.


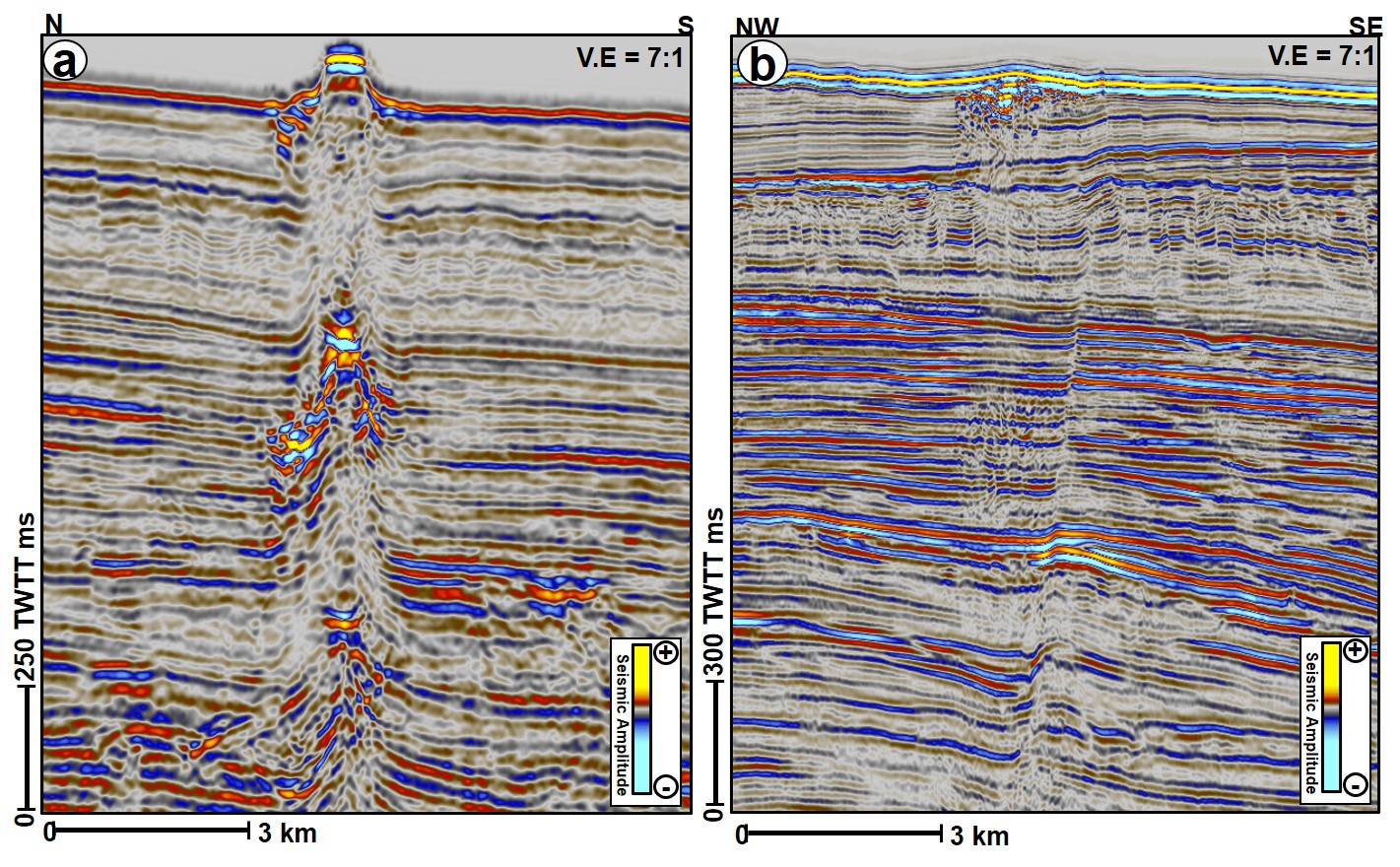


Figure S2: Uninterpreted seismic sections of Figure 6 in the main text.


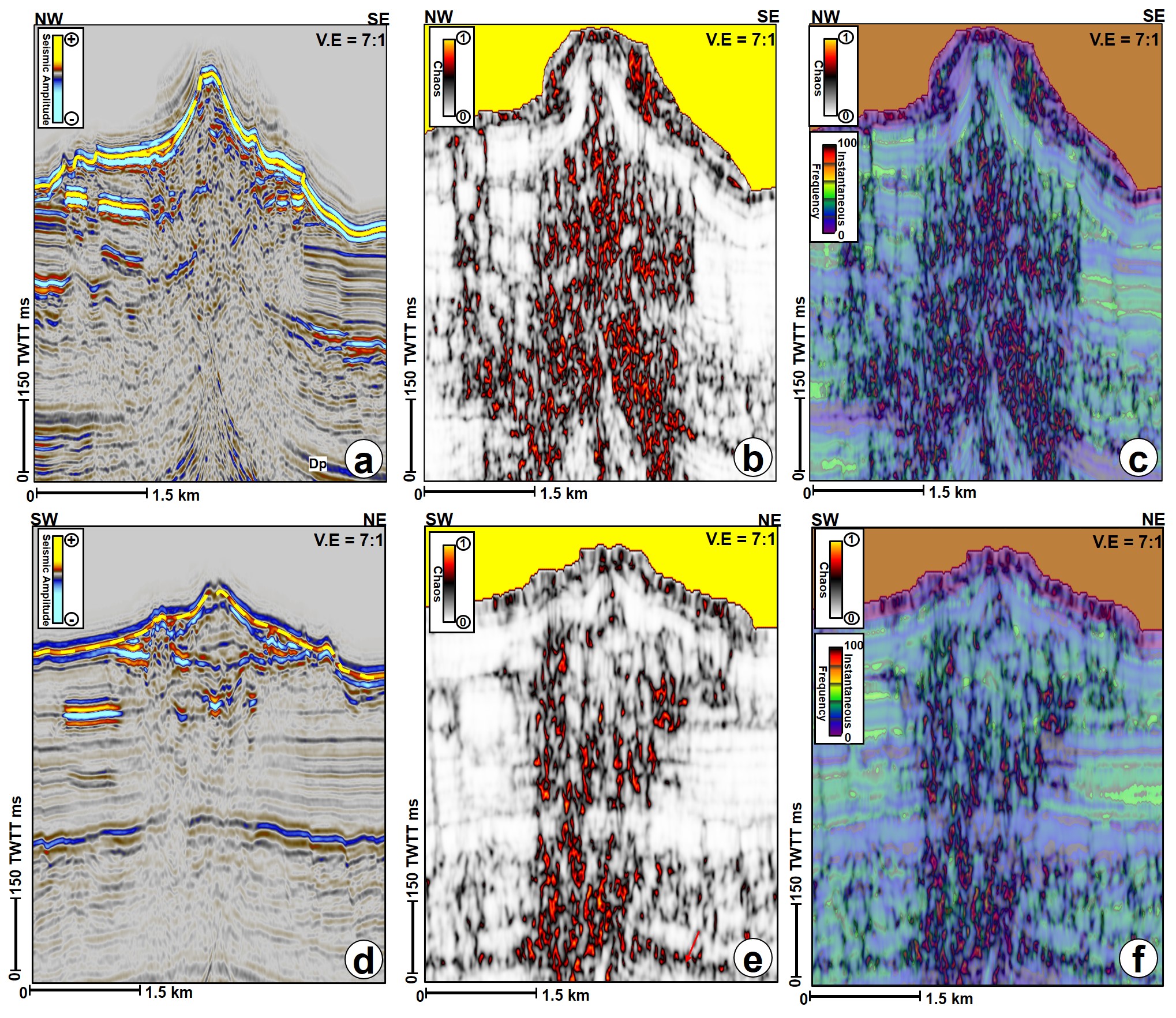


Figure S3: Uninterpreted seismic, chao and instantaneous frequency sections of Figure 8 in the main text.


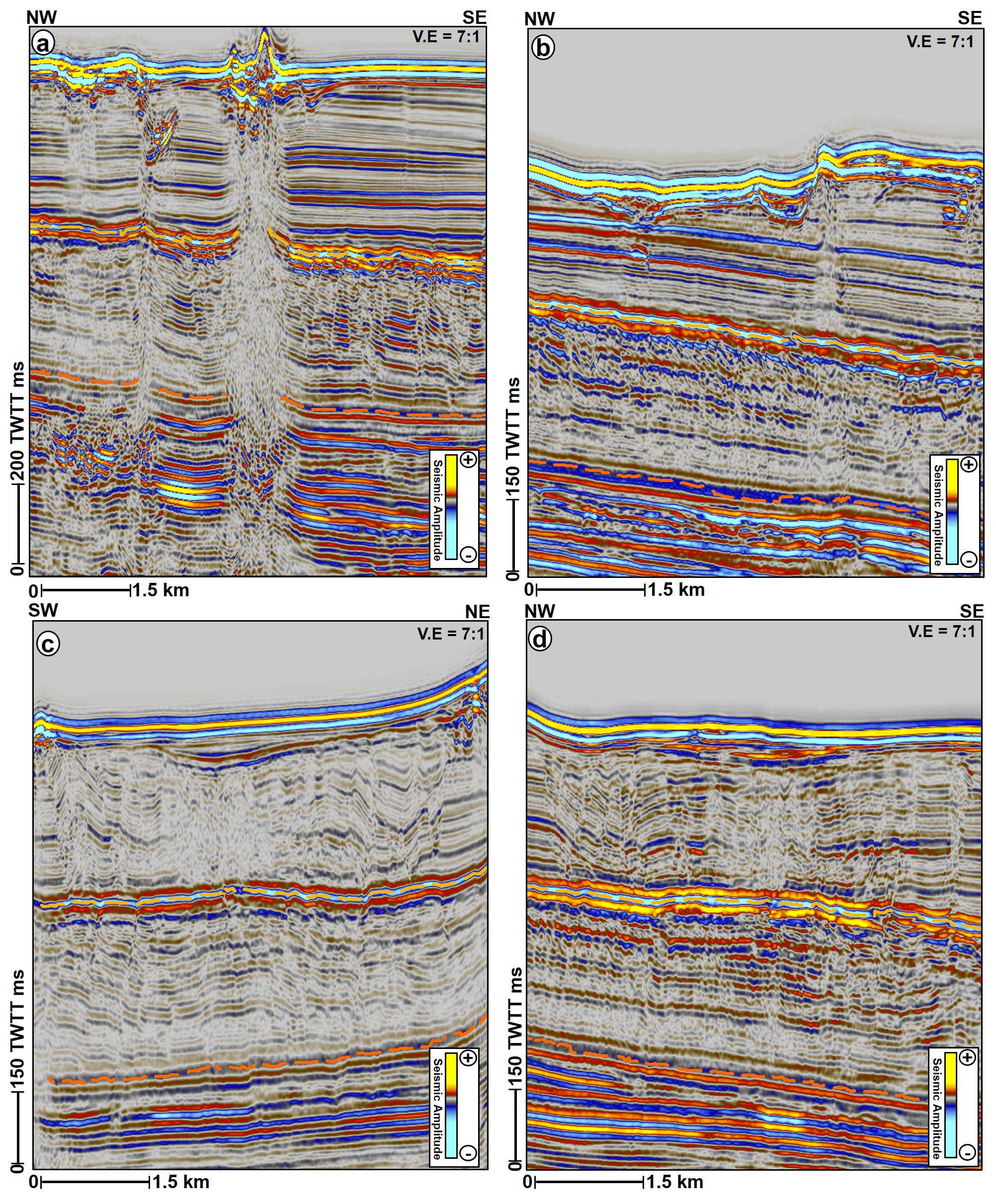


Figure S4: Uninterpreted seismic sections of Figure 9 in the main text.


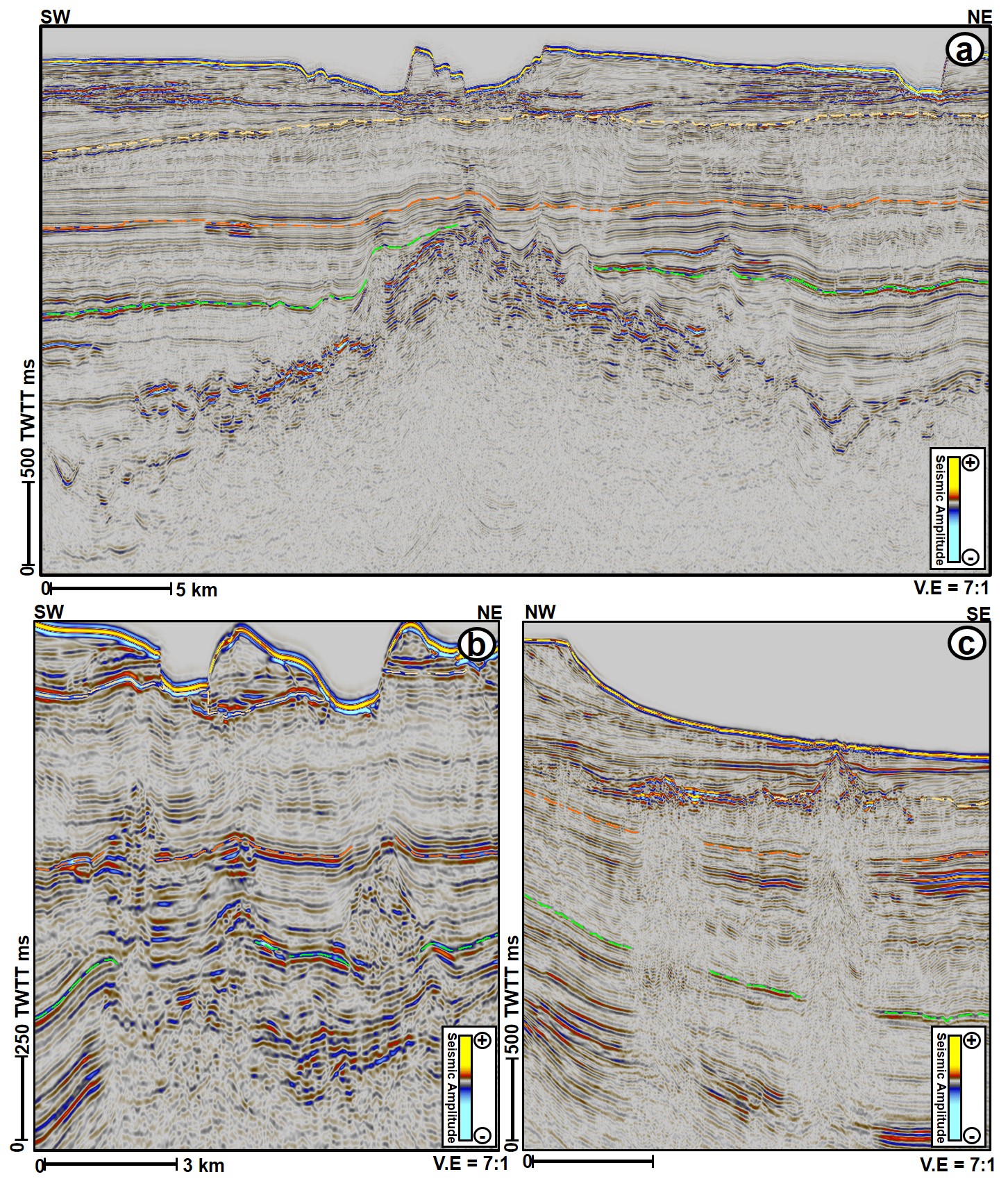


Figure S5: Uninterpreted seismic sections of Figure 10 in the main text.
